# Strengthen or Weaken: Evolutionary Directions of Cross‐Feeding After Formation

**DOI:** 10.1111/1758-2229.70175

**Published:** 2025-08-10

**Authors:** Laipeng Luo, Xiaoli Chen, Bingwen Liu, Yong Nie, Xiao‐Lei Wu

**Affiliations:** ^1^ College of Engineering Peking University Beijing China; ^2^ Institute of Ecology Peking University Beijing China; ^3^ Oil and Gas Research Center Peking University Beijing China

**Keywords:** cross‐feeding, eco‐evolutionary dynamics, evolutionary direction, mutualism

## Abstract

Interactions between species and the evolution of strains are important biotic factors determining the microbial community dynamics, with these two processes being deeply intertwined. Cross‐feeding is a prevailing mutualistic interaction in natural microbial communities in which metabolites secreted by one microbe can be utilised by another. Constructing synthetic microbial consortia based on cross‐feeding is a promising strategy for bioremediation and bioproduction. But how to improve the performance and the stability of consortia remains a challenge. This review discusses the features of two opposite evolutionary directions of cross‐feeding consortia over time, providing insights into the factors affecting the evolutionary process. While coevolving, cross‐feeding may strengthen with stronger metabolic coupling, deeper growth dependence, and/or deeper evolutionary dependence; then the consortia become reinforced. Conversely, unsuitable environmental conditions can lead to the direct collapse of the cross‐feeding consortia due to metabolic decoupling, partner extinction, or cheater dominance. The loss of the fitness advantage and the constraints on the evolutionary ability can also lead to the weakening of cross‐feeding. Cross‐feeding partners can affect the evolution of focal strains from different aspects, such as niche space, selective pressure, horizontal gene transfer, and evolutionary rate. Analysing cross‐feeding from an evolutionary perspective will advance our understanding of microbial community dynamics and enable rational designs of efficient and stable synthetic microbial consortia.

## Graphical abstract

There are two evolutionary directions for cross‐feeding consortia after formation: strengthening and weakening. Simultaneously, cross‐feeding reshapes the focal strain evolution from niche space, selective pressure, horizontal gene transfer, and evolutionary rate.

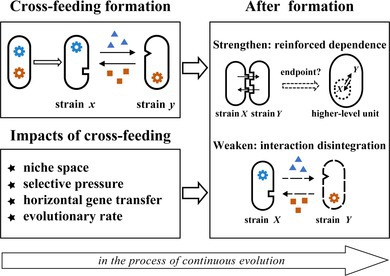

## 1 Introduction

Microorganisms are important components of the ecosystem, contributing to biogeochemical cycles, human health, bioremediation processes, and industrial biotechnology applications. Within microbial communities, there are many types of inter‐specific interactions (e.g., mutualism and competition) formed through direct cell–cell connections or via excreted metabolites (Pande et al. 2015; Kost et al. 2023; Silverstein et al. 2024). Cross‐feeding is a typical mutualistic interaction in which the exchange of metabolites can bring participating species fitness benefits (Smith and Schuster 2024). This interaction is ubiquitous in natural microbial communities and can affect the composition, structure, function, and stability of the communities (Mataigne et al. 2021; Pontrelli et al. 2022). Constructing synthetic microbial consortia is a promising strategy for degrading complex substances and producing target molecules (Sgobba and Wendisch 2020). To date, many synthetic microbial consortia constructed based on cross‐feeding have been successfully used for the degradation of harmful or complex substances, such as *n*‐alkane (Hu et al. 2020) and alginate (D'Souza et al. 2023), the bioproduction of industrially relevant metabolites (Aulakh et al. 2023), and increasing the aluminium tolerance (Ma et al. 2024). The cross‐feeding consortium can also show two‐tiered mutualism to improve the collective metabolic activity and maintain the ecological competitiveness against other soil microbes (Ge et al. 2023). However, some consortia failed due to poor growth at the beginning (Aulakh et al. 2023) or being selected against during evolution (Chen et al. 2024). Knowing in what conditions cross‐feeding is selected for is important to construct synthetic microbial consortia with high and long‐lasting performance.

Evolution is also an important driver of microbial community properties and dynamics (Gorter et al. 2020). For individual strains, the more beneficial phenotypes are favoured by natural selection (Mavrommati et al. 2022). The genotypes with higher fitness can usually be fixed and lead to adaptation to their living environments, while new mutations continuously emerge in the evolutionary process. However, on the one hand, the fitness effect of a mutation is modifiable and influenced by the abiotic and biotic environments (Martinson et al. 2023). Thus, in complex microbial communities, the genotypes with the higher fitness mutation that can be fixed may be altered due to the influence of interaction with other strains or the changing micro‐environments, leading to a different evolutionary trajectory of the focal strains (Lawrence et al. 2012). On the other hand, the newly evolved traits of the focal strains can further accelerate environmental modifications, affecting the evolution of all species existing in this environment (Barraclough 2015). Taken together, the evolutionary process of an interacting consortium may be more complicated due to the eco‐evolutionary feedback.

As for a synthetic microbial consortium constructed based on cross‐feeding, after the formation of interaction, there are two intuitive directions in the evolutionary process: “strengthening” and “weakening”. Here, we reviewed the different features of these two evolutionary directions and the impacts that cross‐feeding has on strain evolution, providing an evolutionary insight into the microbial community dynamics. These will be helpful in designing the synthetic microbial consortia to maintain their stability and improve their function.

## 2 How Does Cross‐Feeding Form

In a cross‐feeding consortium, the metabolites secreted by one strain can be taken up and utilised by another strain, and the exchange of metabolites can promote the growth of both strains. According to the metabolites and beneficial traits, cross‐feeding has different forms, such as by‐product cross‐feeding (where one strain releases a metabolic by‐product that benefits another strain), cooperative cross‐feeding (where one strain actively invests resources to produce metabolites to benefit the interaction partner), and cross‐protection (where one strain secretes extracellular enzymes to detoxicate the toxic metabolites, protecting the interaction partner) (D'Souza et al. 2018; Smith et al. 2019). There is much work studying cross‐feeding formation around two key questions: metabolite externalisation and gene loss. For the former, McKinlay had a comprehensive review of the mechanisms of metabolite externalisation. Communally valuable metabolites can be externalised due to cell envelope metabolism, diffusion across a membrane, mechanosensitive channels and facilitated diffusion, cell lysis, or active externalisation, while the release might maintain homeostatic levels (McKinlay 2023). Also, a “noise‐averaging cooperation” theory had been put forward, demonstrating that metabolite leakage can increase collective fitness. In this theory, bacteria are prone to noisy regulation of metabolism, which limits their growth rate, while related bacteria can share metabolites to “average out” noise and improve their collective growth (Lopez and Wingreen 2022). With the available metabolites in the environment, strains with gene loss seem to be able to survive. Gene loss may occur before coculture; then the auxotrophy is rescued by cross‐feeding (Chuang et al. 2024). It may also be favoured by natural selection after coculture according to the Black Queen Hypothesis (BQH): when certain microbes secrete useful public goods into the environment, neighbouring cells can gain fitness advantages by using these shared resources rather than synthesising them by themselves, ultimately leading to functional gene loss (Morris et al. 2012). As for microbes with a symbiotic lifestyle, repeated bottlenecks of relatively small populations may result in a reduction in genome size and a concomitant loss of essential genes, driven by genetic drift (D'Souza et al. 2014).

Cross‐feeding can transform between different forms. In the review of D'Souza et al., a hypothetical model had been used to explain the evolution of cooperative metabolic cross‐feeding (D'Souza et al. 2018): (1) microbial cells grow autonomously and inevitably release metabolites into the extracellular environment, forming an exometabolome, (2) mutants that lose the ability to synthesise some metabolites arise, and these auxotrophs can grow by consuming externally available metabolites, thus forming unidirectional by‐product cross‐feeding, (3) two auxotrophs survive depending on the metabolites released by each other, which means that bidirectional by‐product cross‐feeding is established, (4) one partner can benefit from unilaterally increasing its metabolite production because it increases the amount of metabolites it can receive in return, showing the by‐product reciprocity, and (5) bidirectional cooperative cross‐feeding emerges when each of the involved organisms starts to actively invest resources into metabolite production to benefit the respective partner.

## 3 Evolutionary Directions of Cross‐Feeding After Formation

After cross‐feeding formation, the consortia will still keep changing, driven by the eco‐evolutionary dynamics (Martiny et al. 2023). How will cross‐feeding change in the evolutionary process? Here, we focused on the ecological and evolutionary features of cross‐feeding partners while evolving: if the dependence between partners increases, we call it the “strengthening” of cross‐feeding, and if the dependence decreases, we call it the “weakening”.

### 3.1 Strengthen—Reinforced Dependence

**FIGURE 1:**
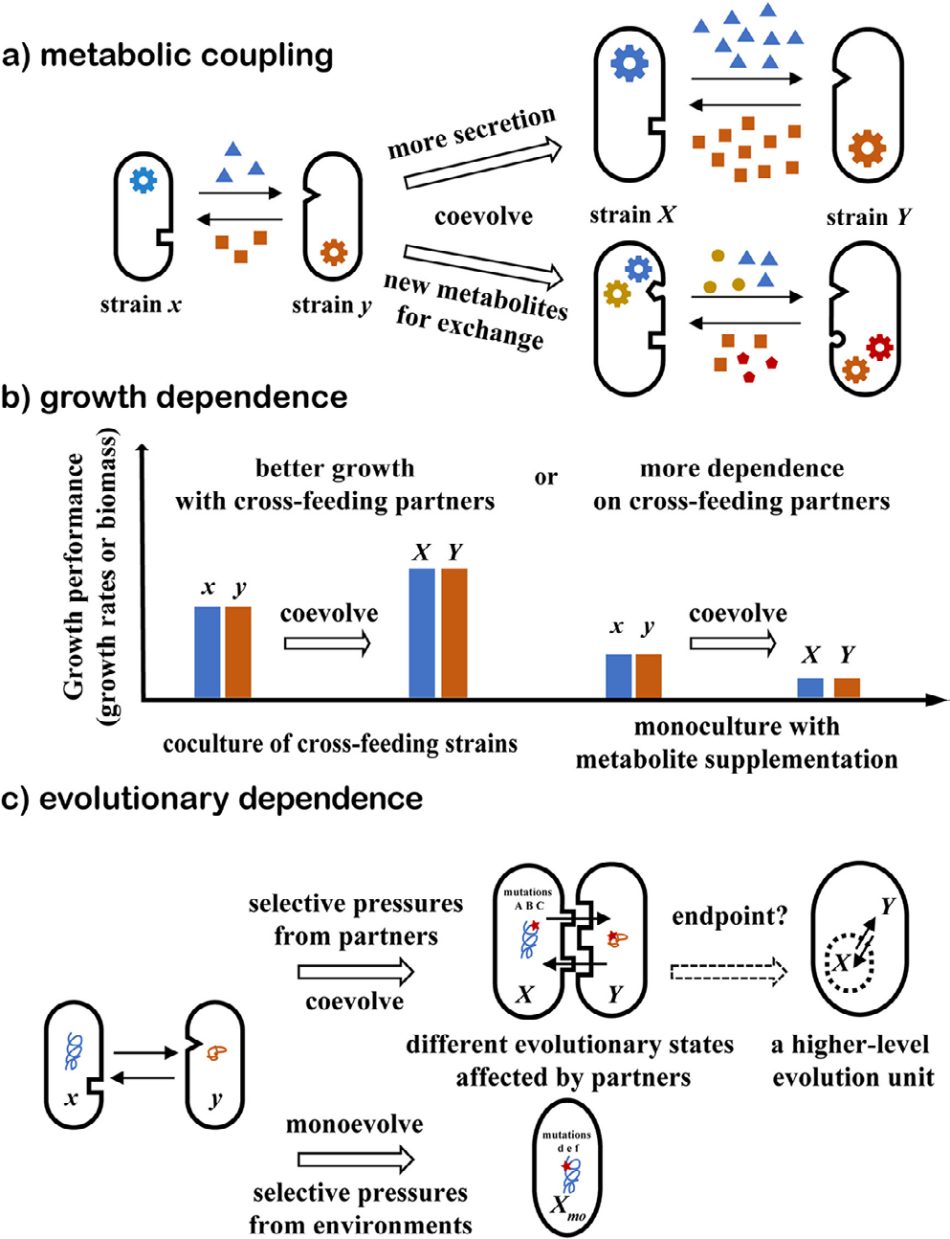
Strengthening of cross‐feeding. (a) Metabolic coupling becomes stronger. After coevolving, the increased secretion of exchanged metabolites and the newly formed metabolite exchange show the strengthening of cross‐feeding. (b) Growth dependence becomes deeper. After coevolving, the growth performance (growth rates or biomass) of the coculture of cross‐feeding strains increased; but sometimes the growth performance of monoculture with metabolite supplementation decreased, showing more dependence on cross‐feeding partners. (c) Evolutionary dependence becomes deeper. Coevolution enables the strain to reach a different evolutionary state due to the different selected mutations affected by cross‐feeding partners. Whether cross‐feeding consortia can transition to a higher‐level evolutionary unit remains a question. Strains *X*, *Y* evolved from strains *x*, *y*, respectively.

In some cases, the ecological and evolutionary dependence between cross‐feeding partners gradually increases in the evolutionary process; that is, a closer interaction relationship is forming. The “strengthening” of cross‐feeding can be seen from the stronger metabolic coupling, the deeper growth dependence, and the deeper evolutionary dependence (as shown in Figure 1).

#### 3.1.1 The Stronger Metabolic Coupling

The exchange of metabolites is the basis of cross‐feeding. The cross‐feeding partners are coupled due to metabolite dependence, and the metabolic pathway steps are distributed across different strains. For a cross‐feeding consortium, it is a sign of “strengthening” that the more metabolites or new metabolic pathways are involved in cross‐feeding.

The increased secretion of metabolites for exchange is one manifestation of the strengthening of cross‐feeding. When the strains exchange metabolites and thus gain growth advantages, the cross‐feeding is established. However, the yield of the metabolites is usually not high. Initially, the metabolites may be only available because of “accidental” leakage, but active export processes may evolve later (Preussger et al. 2020; McKinlay 2023). With coevolution, if the strains secrete more metabolites, this is a sign of cross‐feeding becoming strengthened. Although secreting more metabolites may be an energy cost for the producer, it can enhance the growth of cross‐feeding partners, to obtain more benefits produced by partners in return (Marchal et al. 2017; Harcombe et al. 2018). If the energy saved by enhancing the partner's growth exceeds the cost of the increased metabolite export, then enhanced export will be selected (Pande et al. 2014). Through positive feedback, the yield of both metabolites exchanged between strains can reach a higher level (Preussger et al. 2020). Using this approach, we can design cross‐feeding microbial consortia coevolving in certain laboratory conditions to obtain higher‐yield functional strains for target metabolites. For example, Konstantinidis et al. coevolved the obligate cross‐feeding consortia consisting of lactic acid bacteria auxotrophic for amino acids and engineered *Saccharomyces cerevisiae* auxotrophic for B‐group vitamins (riboflavin or folate). After coevolution, lactic acid bacterial isolates with enhanced secretion of riboflavin and folate were obtained, and further analyses showed altered metabolic regulation towards increased supply of the vitamin precursors in the evolved lactic acid bacteria (Konstantinidis et al. 2021). Harcombe constructed a two‐species consortium involving *Salmonella enterica* and an *Escherichia coli* mutant unable to synthesise methionine. In lactose minimal medium, *S. enterica* consumed metabolic waste from *E. coli* and evolved to excrete higher levels of methionine, about an approximate 15‐fold increase compared with the wild type (Harcombe 2010).

The increased fitness of more secreted metabolites can be further explained by the law of comparative advantage and the economies of scale, which are also fundamental principles driving the economics in human society (Adkins‐Jablonsky et al. 2021). The advantage of metabolite exchange comes from production specialisation with higher efficiency and increased production with lower per‐unit energy costs. In detail, two trading strains can produce two metabolites at different production efficiencies. When strains specialise in producing metabolites where they have higher efficiency (i.e., with comparative advantage) and trade with partners, community‐level metabolic efficiency increases. After specialisation, the average energy cost per molecule of metabolite decreases while the total production increases because there is a fixed cost for the metabolic pathway (Tasoff et al. 2015; Huelsmann et al. 2024). So, the increased secretion of metabolites for exchange is profitable. Analogous to human society, the microbial exometabolome, composed of all secreted metabolites, can be seen as a market in which the cross‐feeding strains can meet their needs (Werner et al. 2014; Douglas 2020). Such an exchange can enable them to gain additional benefits.

The coupling of broader metabolic pathways is another manifestation of the strengthening of cross‐feeding. The exchange of metabolites bridges the metabolic pathways of cross‐feeding strains, establishing metabolic dependence. If the cross‐feeding consortium starts to exchange new metabolites in addition to the initial metabolites, the dependence between the strains increases. Then, the evolved cross‐feeding consortium will have a more complex structure of coupled metabolic pathways, showing stronger metabolic coupling. According to the Black Queen Hypothesis, gene loss can be evolutionarily favoured when enhancing fitness (Morris et al. 2012). So, when the interacting strains have metabolic functional redundancy, it is still possible for cross‐feeding to form new metabolite exchange, resulting in further function specialisation (Smith et al. 2019). However, the effect of losing multiple metabolic genes on strain fitness is not clear, and the evolutionary endpoints under BQH dynamics remain unpredictable (D'Souza et al. 2015; Smith et al. 2019).

Several studies showed that new coupled metabolic pathways can appear in the evolutionary process due to further gene loss. D'Souza et al. serially propagated replicate populations of *Escherichia coli* in amino acid‐containing environments and found that auxotrophic genotypes rapidly evolved in less than 2000 generations. The number of amino acid auxotrophies identified per isolated strain gradually increased, reaching an average of 10 amino acids per strain after 2000 generations, while some strains needed all 20 amino acids for growth (D'Souza and Kost 2016). Wang et al. built an individual‐based model (IBM), where an autonomous population could produce three essential public goods, and the functions were allowed to lose randomly to study how reductive evolution shaped microbial interdependencies. In the simulations, seven new genotypes evolved, including three one‐function loss genotypes, three two‐function loss genotypes, and one three‐function loss genotype, inferring a complex cross‐feeding network and the increasing genotype interdependence (Wang et al. 2021). The metabolite availability seems to be important for the loss of more functions. Auxotrophs may gain a fitness advantage when utilising metabolites in the environment and then be favoured. Also, the broader exchanges can emerge from the extra metabolite secretion. The extra secretion may be selected for if the benefits of cooperation outweigh the costs; that is, the producer increases the growth of cross‐feeding partners and gains more return. For example, Harcombe et al. transferred a synthetic cross‐feeding consortium where *Salmonella enterica* secreted methionine in exchange for acetate generated by methionine auxotrophic *Escherichia coli* and found that *E. coli* evolved to secrete costly galactose. Galactose secretion was favoured because it enhanced cooperation with the cross‐feeding partner. By secreting galactose, *E. coli* increased the abundance of *S. enterica* , thereby increasing the production of methionine. The preferential access of galactose producers to the additional methionine was afforded by the spatial structure (Harcombe et al. 2018).

#### 3.1.2 The Deeper Growth Dependence

Another characteristic of cross‐feeding can be the improved growth performance of each cross‐feeding subpopulation. The reciprocal cross‐feeding of metabolites can accelerate growth (Dutta and Saini 2021), broaden the substrate spectrum (Oña et al. 2021), and alleviate the toxic burden (Lee et al. 2021). By comparing the growth performance (growth rates or biomass) of cross‐feeding strains in monoculture and coculture, the growth dependence between strains can be seen (Liu et al. 2019). For example, if both auxotrophs can grow in coculture but cannot grow in monoculture without supplied metabolites, they show obligate growth dependence (Pande et al. 2014). Furthermore, by comparing the growth performance of evolved strains with that of ancestral strains in monoculture, the degree of adaptation of evolved strains to the mutual lifestyle can be inferred.

The growth performance of cross‐feeding consortia can be better after coevolving. For a cross‐feeding consortium, the partners not only provide the metabolites for exchange but also become a part of the biotic environment with new selective pressure, affecting the costs and the benefits experienced by each subpopulation (Vidal and Segraves 2021). In the beginning, cross‐feeding strains may be maladaptive to each other, competing for non‐exchanged metabolites or secreting and uptaking substances inefficiently, especially when the consortium is constructed by two engineered strains without any coevolutionary history (Harcombe 2010; LaSarre et al. 2017; Vidal and Segraves 2021). In the evolutionary process, the mutants that can enhance metabolite acquisition, reduce negative interactions, or promote partner growth may increase the benefits of metabolite exchange and then be favoured (Fritts et al. 2020; Chacón et al. 2021; Venkataram et al. 2023). Then, the growth performance of the cross‐feeding consortium may be better than the initial coculture due to the adaptation of the strains to their partners. Zhang et al. constructed a synthetic cross‐feeding consortium comprised of leucine and lysine *Escherichia coli* auxotrophs and found that the consortium gained increased growth rates and biomass yields after coevolution. The evolved strains could also increase the growth performance when cocultured with unevolved parental strains, implying that the evolved isolates likely had increased uptake and/or release of leucine or lysine (Zhang and Reed 2014). Similarly, Zuchowski et al. generated a stable synthetic cross‐feeding consortium with arginine and leucine auxotrophic *Corynebacterium glutamicum* . After adaptive laboratory evolution, the consortium gained a 23% growth rate increase, and the efficient amino acid export and uptake of evolved strains appeared to be one of the key factors for the improved consortium growth (Zuchowski et al. 2023).

The evolved strains may be more adapted to the mutual lifestyle. After coevolving, the strains may be more suitable for the mutual lifestyle and more dependent on their partners. That means the evolved strains may grow worse than the ancestral strains in monoculture, although supplied with exogenous essential metabolites. This phenomenon has been observed in some evolution experiments. Zhang et al. found that although evolved isolates gained increased growth in coculture, they exhibited decreased growth in monoculture compared with the unevolved strains with leucine or lysine supplementation (Zhang and Reed 2014). Preussger et al. explained that the trade‐off was due to the costly adaptation to coevolved partners (Preussger et al. 2020). They found that after the coevolution of a cooperative cross‐feeding consortium composed of two *Escherichia coli* amino acid auxotrophs, the auxotrophs significantly increased their amino acid production levels. In coculture, the benefits from partners could cover the costs, but in monoculture, the investment had no return, resulting in decreased growth. However, if the evolved strains can obtain benefits from the environment, growth in nutrient‐rich monoculture can also increase, such as the evolved *E. coli* with enhanced ammonium uptake ability (Fritts et al. 2020). What's more, the cross‐feeding strains may rewire the metabolic network to adapt to the mutual lifestyle. For example, Scarinci et al. found that the cooperative cross‐feeding between auxotrophs of *Escherichia coli* and *Saccharomyces cerevisiae* had been strengthened after the experimental coevolution. The evolved yeast strains gained additional dependence on the bacterial partner, using arginine provided by *E. coli* as the primary nitrogen source rather than ammonium (Scarinci et al. 2024). The emergence of de novo interdependence between partners for nitrogen metabolism showed the “strengthening” of cross‐feeding.

#### 3.1.3 The Deeper Evolutionary Dependence

Cross‐feeding may change the strains' evolutionary trajectories. The evolutionary states accessible to strains can be affected by the interacting partners (Bottery et al. 2021; Pearl Mizrahi et al. 2023). Compared with the strains evolving in monoculture, the focal strains in cross‐feeding consortia may evolve different traits along another evolutionary trajectory. We define evolutionary dependence as the extent to which cross‐feeding partners affect the evolution of the focal strains, which can be seen from the different favoured mutations between coevolution and monoevolution. The more traits evolved under the influence of cross‐feeding partners, the deeper the evolutionary dependence becomes. The deeper evolutionary dependence may be associated with the stronger metabolic coupling and/or deeper growth dependence mentioned above because the different mutations may be the genetic basis of ecological adaptation to the cross‐feeding partners. For example, the mutation of *E. coli* in galactokinase (*galK*) was the reason for the secretion of costly galactose while coevolving with *S. enterica* . The mutation was favoured due to selection for mutualistic benefits. However, it resulted in a lower growth rate and yield in monoculture, likely selected against without the cross‐feeding partners (Harcombe et al. 2018). Zuchowski et al. also found that several mutations increased growth in coculture but did not result in better growth of a monoculture supplemented with the required amino acid, implying different evolutionary trajectories of the focal strains in the presence and absence of the cross‐feeding partners (Zuchowski et al. 2023). Also, cross‐feeding can affect the evolutionary response of strains to environmental stress, such as antibiotics, showing the deeper evolutionary dependence (Adamowicz et al. 2020; Durand et al. 2023). Adamowicz et al. experimentally evolved a synthetic microbial consortium consisting of *Escherichia coli* and *Salmonella enterica* in coculture and monoculture controls along gradients of two different antibiotics, rifampicin and ampicillin. The authors found that different resistance mechanisms arose in coevolution and monoevolution under ampicillin selection. In particular, mutations in an essential cell division protein, FtsI, arose in *S. enterica* only in coevolution (Adamowicz et al. 2020).

Can cross‐feeding lead to major transitions in individuality? What is the endpoint of the “strengthening” of cross‐feeding? An interesting question is whether mutualistic communities can transition into evolutionary individuals (Salazar and Mitri 2025). Some work studied the transition from lower to higher‐level evolutionary units. Major evolutionary transitions theory proposes that cooperative systems may evolve into functionally integrated units, where previously independent cells coordinate reproduction and metabolic tasks (Szathmary 2015; West et al. 2015). Obligate endosymbiosis may represent a major evolutionary transition in individuality, in which distantly related species integrate to form a single replicating individual (Rafiqi et al. 2020). The endosymbiotic theory is also usually used to explain the mitochondrial and chloroplast establishment in eukaryotes (Zachar and Boza 2020). There seem to be consortia with higher‐level structures in which multiple microorganisms are closely linked by interactions, such as lichens and biofilms, showing the possibility of transition (Carr et al. 2021; Libby and Ratcliff 2021). Lichens are important symbionts that mainly consist of fungi and algae or cyanobacteria. Biofilms are defined as an aggregation of microbes that can attach to a surface via extracellular products. Both of them show obvious collective growth, that is, the components specialise in performing different tasks and interact with each other through direct contact or metabolite communication and may form a uniquely complex 3‐D structure (Carr et al. 2021). However, it is still controversial whether they are a higher‐level evolutionary unit or just an aggregate of individuals. To describe the individuality of collective consortia, Salazar and Mitri proposed a framework where evolutionary individuality was defined by how much a collective exhibits positive interactions, functional integration, and entrenchment. As for cross‐feeding consortia consisting of mutualistic auxotrophs, there seemed to be relatively high positive interactions and functional integration, but low entrenchment (Salazar and Mitri 2025). The way to increase the individuality of microbial communities is still unexplored.

### 3.2 Weaken—Interaction Disintegration

**FIGURE 2:**
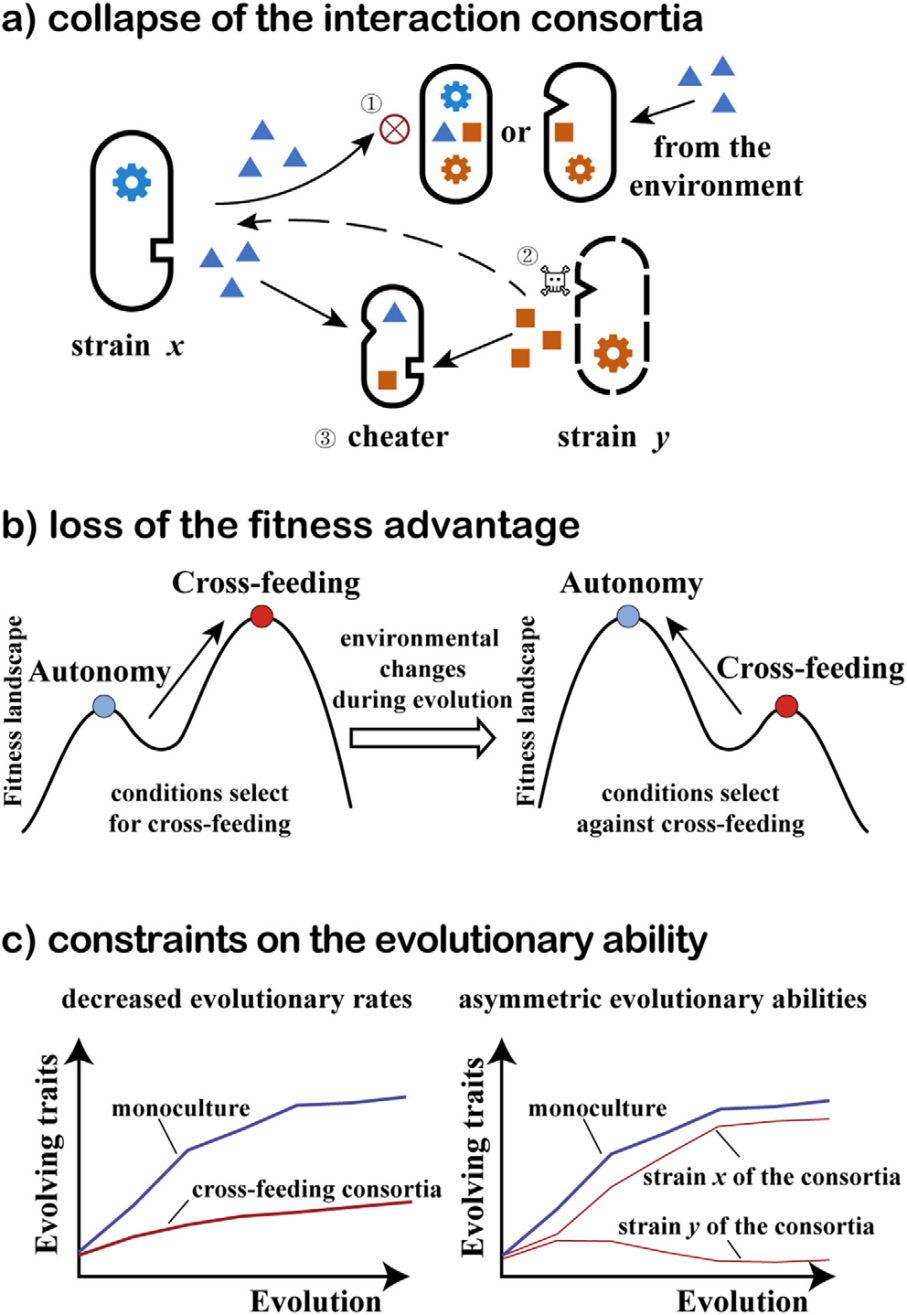
Weakening of cross‐feeding. (a) The cross‐feeding consortia collapse directly. (1) The metabolites for exchange are no longer needed because of the regain of lost functional genes or the rich metabolites from the environment. (2) The partners are extinct due to the small population sizes or unsuitable environmental conditions. (3) The cheaters rob the public goods, leading to the overexploitation of shared metabolites and the collapse of cross‐feeding consortia. (b) The fitness advantage of the strains with cross‐feeding genotype is lost because the environmental conditions select against cross‐feeding during evolution. In such conditions, genotypes of other metabolic strategies have higher fitness, and the strains evolve to climb the peak of the fitness landscape, resulting in a shift of metabolic strategies. (c) The evolutionary ability of cross‐feeding consortia has been constrained due to the decreased evolutionary rates of the strains or the asymmetric evolutionary abilities between the interacting strains.

Studies also demonstrate that the ecological or evolutionary dependence between cross‐feeding partners may be gradually weakened in the evolutionary process, potentially culminating in complete interaction collapse. The “weakening” of cross‐feeding can be classified into three forms, which are not mutually exclusive: the collapse of the interaction consortia, the loss of the fitness advantage, and the constraints on the evolutionary ability (as shown in Figure 2). The cases may meet more than one criterion.

#### 3.2.1 The Collapse of the Interaction Consortia

As a context‐dependent interaction, cross‐feeding can be affected by many factors. This interaction relies on sustained metabolite exchange between specialised phenotypes. Thus, at any point during evolution, the consortia may collapse if the exchanged metabolites are no longer needed, the partners are missing, or the exchange cannot be implemented.

##### 3.2.1.1 The Dependence on Metabolites Disappears

The cross‐feeding consortium is constructed based on metabolic dependence. The addition of exchanged metabolites will reduce this dependence and then affect the ecological stability and evolutionary trajectory of the consortium (Hammarlund et al. 2019; Zhao et al. 2023). Generally, nutrient‐poor environments increase the likelihood that cooperative interactions arise. For natural communities, cooperation helps to reduce dependence on the abiotic environment and occupy more diverse habitats with resilience to nutrient changes (Machado et al. 2021). When experimentally evolved, cross‐feeding is selected for in a nutrient‐poor environment but selected against in a nutrient‐rich environment (Murillo‐Roos et al. 2022). Once the environment is rich enough and the strains can obtain all necessary metabolites directly from the environment, cross‐feeding is likely to be disadvantageous. The secretion of strains is selected against, and then the cross‐feeding interaction degrades. When the cross‐feeding consortia that evolved in the nutrient‐rich environment switched to the nutrient‐poor environment, the lower growth of the evolved consortia was observed (Murillo‐Roos et al. 2022). In a cellular automaton model, CELL‐ABC (Cellular Automaton of Bacterial Cross‐feeding), it was also found that amino acid supplementation to the environment decoupled obligate cross‐feeding and eliminated the requirements for reciprocal cross‐feeding. Auxotrophic cells that saved amino acid production costs were generally favoured over the metabolically autonomous prototrophs and cross‐feeders that were auxotrophic for one amino acid, yet produced and released increased amounts of the respective other amino acid into the cell‐external environment (Germerodt et al. 2016).

Cross‐feeding may also break down if the strains do not need the metabolites provided by partners in the evolutionary process. The strains may rewire the metabolic networks to avoid dependence on the exchanged metabolites, evolve new traits to compensate for the lost function, or regain lost functional genes (Chen et al. 2024; Piccardi et al. 2024; Melero‐Jiménez et al. 2025). Once the strains obtain the full capacity to survive without the metabolites from the partners, the metabolic dependence disappears. For example, Melero‐Jiménez et al. constructed an obligate cross‐feeding consortium consisting of methionine and isoleucine auxotrophic *Escherichia coli* . The consortium was propagated under no stress, salinity stress, and p‐nitrophenol stress. The authors found that only one of the strains survived by metabolically bypassing the auxotrophy due to some new mutations in genes involved in amino acid biosynthesis under either stress, since the prototrophic strain was less affected by either salinity or p‐nitrophenol (Melero‐Jiménez et al. 2025). Similarly, Chen et al. found that after evolution in fluctuating environments, autonomous genotypes evolved from two cooperative specialists, and the knock‐out genes were potentially regained by horizontal gene transfer. In the fluctuating condition where naphthalene and pyruvate were alternately supplied in the passaging cycles, the two specialists alternately engaged in mutualism or competition in different cycles. The increased coexistence of both specialists and higher cell density in the pyruvate cycles, and the higher growth rate and biomass yield of autonomists in the naphthalene cycles were responsible for the evolution of autonomy (Chen et al. 2024).

##### 3.2.1.2 The Interaction Ceases Because of the Extinction of Partners

Cross‐feeding is a group‐level interaction existing between different phenotypes. Its stability hinges on maintaining critical population thresholds for both partners. Population dynamics are important features to capture the changes in population sizes of different strains in the evolutionary process and could potentially be used to predict community stability (Bull and Harcombe 2009; Van den Bergh et al. 2018). Bull and Harcombe put forward that population dynamics constrain the cooperative evolution of cross‐feeding. They found that the benefit of cooperative cross‐feeding applied only in the range of intermediate cell densities (Bull and Harcombe 2009). If the abundance of one or both phenotypes becomes too low to maintain growth, the consortium will go to extinction (Hillesland and Stahl 2010). The Allee effect provides an explanation by a decreased fitness at low densities so that the individual growth rate reaches a maximum at an intermediate density (Vet et al. 2020). That means there is a density threshold for survival, and populations below the threshold face extinction risks (Sanchez and Gore 2013). Also, large populations may consume lots of resources, leading to environmental deterioration. Exceeding the upper limit is also detrimental to the stability of the community (Bull and Harcombe 2009).

Environmental conditions are another important factor influencing cross‐feeding consortium stability. Strain growth is affected by many abiotic factors such as temperature, pressure, pH, and antibiotics. Different strains prefer different growth conditions, so the niche space of the cross‐feeding consortium will be contracted to ensure the growth of all different phenotypes (Malard and Guisan 2023). The weakest‐link hypothesis is an example that the niche space of obligate cross‐feeding coculture is contracted. Resistant bacteria were inhibited by lower concentrations of antibiotic when cross‐feeding than when growing independently (Adamowicz et al. 2018; Adamowicz and Harcombe 2020). What is more, environmental conditions always change, and strains can also modify the environment. However, the environmental change is not always profitable for all strains. Participating strains in the consortium may die out because the environmental conditions are no longer suitable (Ratzke and Gore 2018). For example, the soil bacteria *Paenibacillus* sp. can secrete a variety of organic acids, leading to environmental acidification during bacterial growth. The pH could be changed to 4, where the bacteria suddenly started to die. The species induced the low pH values that were detrimental for their own growth and thus harmed themselves, known as ecological suicide (Ratzke et al. 2018). So, if the environmental conditions are not suitable for all strains or change to harmful states, the cross‐feeding consortium may collapse due to the extinction of cross‐feeding partners.

##### 3.2.1.3 The Cooperative Consortia Face the Challenge of Cheaters

Microbial communities are open systems where strains may evolve new phenotypes or other strains can come in. These new phenotypes or strains may influence the interactions between original strains and even change the interaction network. For cross‐feeding, cheaters are genotypes that are evolutionarily derived from a cooperative partner, showing “selfish” behaviour by taking benefit from the public goods produced by other members, without paying the costs (Sun et al. 2019; Chomicki et al. 2020). For example, pyoverdine non‐producers were cheaters that enjoyed a large fitness advantage in coculture with producers and could spread to high frequency when limited by carbon (Sexton and Schuster 2017). Over evolutionary time, selection for cheating threatens mutualism persistence (Strassmann et al. 2000; Velicer et al. 2000; Ghoul et al. 2014). The invasion of cheaters into cooperative consortia has been extensively studied to assess the stability of cooperative consortia. The cooperative consortia seem to have some anti‐cheater strategies to defend against cheaters, such as local interactions, self‐organised spatial patterns, or reaching a viable equilibrium (Sanchez and Gore 2013; Stump et al. 2018). The cooperation‐independent cheater growth is also important for the population dynamics of microbial cross‐feeding. van Tatenhove‐Pel et al. found that when the growth of cheaters completely depended on cooperators, cooperators outcompeted cheaters. However, cheaters outcompeted cooperators when they could independently grow to only 10% of the consortium's carrying capacity (van Tatenhove‐Pel et al. 2021). The accumulation of cheaters likely poses a serious burden on shared resources, threatening the stability of the consortia (Finn et al. 2022). In a word, the cross‐feeding consortia face the challenge of cheaters in the evolutionary process. If the cross‐feeders cannot control the abundance of cheaters, the overexploitation of shared metabolites may destroy the consortia.

#### 3.2.2 The Loss of the Fitness Advantage

Fitness landscape is a useful concept for the study of evolution by mapping genotypes to fitness. In a fitness landscape, evolution can be seen as “walks” and adaptation as “climbs” to higher positions on the fitness surface (de Visser and Krug 2014; Fragata et al. 2019). Typically, genotypes at the peak of the fitness landscape exhibit the greatest fitness advantage and are favoured in the evolutionary process. Genotypes adapted to different lifestyles have different positions on the fitness surface, and strains always climb to higher positions with fitness advantage. Also, many factors can affect the shape of the fitness landscape, although the mechanism may be unclear. As for strains in cross‐feeding consortia, the conditions that maintain the cross‐feeding genotype at the higher fitness position remain poorly understood. For example, what kinds of metabolites are beneficial when exchanged cannot be precisely speculated. San Roman et al. predicted that cross‐feeding of 58 carbon sources could emerge in the same environment through genome‐scale metabolic modelling, but only cross‐feeding of acetate and glycerol had been experimentally observed (San Roman and Wagner 2020). Meanwhile, unexpected metabolite exchange emerged in some experiments, showing the advantage of cross‐feeding (Harcombe et al. 2018). Mathematical efforts tried to parameterise the conditions to search the parameter space that could result in an increase in fitness with certain assumptions (Lundh and Gerlee 2013). However, how a set of environmental conditions determines the fitness landscape of strains is still unexplored and needs further investigation.

Nevertheless, there is a consensus that the strains tend to choose the metabolic strategy with a higher fitness in the fitness landscape. Cross‐feeding is thought to be costly to maintain and may be selected against if benefits diminish (Weinbach et al. 2022). In conditions that are not suitable for the mutualistic consortia, cross‐feeding is not the peak in the fitness landscape; then the strains in cross‐feeding consortia may change to another metabolic strategy with a fitness advantage. It has been observed that metabolic autonomy can emerge from the interaction consortia as another metabolic strategy (Dragos et al. 2018; Meijer et al. 2020; Pauli et al. 2022). Cross‐feeding and autonomy have different metabolic characteristics and advantages. Autonomy can gain benefits by removing reliance on other strains that may not be encountered in conditions where exchanges are inefficient or public goods are privatised (Oliveira et al. 2014; Lindsay et al. 2019). Cross‐feeding can improve metabolic efficiency and reduce the dependence on external nutrients because of the metabolic specialisation and metabolite exchange (Smith and Schuster 2024). The metabolic strategy shift is determined by the relationship between the microbial consortia and the environment. The metabolic strategy that can provide a higher fitness in certain environmental conditions is located at a higher position in the fitness landscape; and then the strains may evolve towards the corresponding genotype, driven by natural selection. There are lots of studies showing that the metabolic strategy can shift from autonomy to cross‐feeding (Giri et al. 2022). Recently, the reverse process has also been studied. Strains in cross‐feeding consortia without a fitness advantage may switch to autonomous genotypes. Chen et al. found that autonomous genotypes could evolve from two cooperative specialists and dominate the microbial consortia in fluctuating environments, where autonomous genotypes had a higher fitness (Chen et al. 2024). Melero‐Jiménez et al. also found that one strain evolved to bypass the auxotrophy under stress, where the prototrophic strain was less affected and could grow better (Melero‐Jiménez et al. 2025).

#### 3.2.3 The Constraints on the Evolutionary Ability

Microorganisms can adapt to a changing environment through evolution. After metabolic coupling, the ability of cross‐feeding consortia to adapt evolutionarily to new environmental conditions may differ from that of autonomously growing strains. Generally, energy investment in one trait may limit the optimisation of others, showing trade‐offs due to physiological and proteome constraints (Zhu and Dai 2024). The evolutionary ability of a strain to explore the fitness landscape intrinsically depends on its mutation rate, population size, the degree of maladaptation, and the malleability of its genome. However, the intrinsic factors may also be affected by the extrinsic biotic factors (Scheuerl et al. 2020). The constraints on the evolutionary ability of consortia might come from the worse evolutionary ability of the strain due to the biotic interactions, such as lower evolutionary rates, or the asymmetric evolutionary abilities between the interacting strains, resulting in the collapse of cross‐feeding consortia because the strains could not adapt to the rapid environmental changes as a whole (Adamowicz et al. 2020; Pauli et al. 2022; Ye et al. 2025).

For example, Pauli et al. examined how obligate cooperative mutualisms could affect the ability of strains to adapt evolutionarily to changing environmental conditions using a cross‐feeding consortium of two amino acid auxotrophic genotypes of *Escherichia coli* adapted to antibiotic stress, and found that metabolically interdependent strains were generally less able to adapt to environmental stress than autonomously growing strains. The consortia went extinct or grew worse during evolution, inferring that the limited evolutionary ability might be a significant cost that strains had to pay when entering into an obligate mutualistic cooperation (Pauli et al. 2022). Also under antibiotic stress, Adamowicz et al. used a cross‐feeding consortium consisting of *Escherichia coli* and *Salmonella enterica* to study how microbial interactions influence the evolution of resistance. In both rifampicin and ampicillin treatments, the authors observed that resistance evolved more slowly in obligate cocultures than in monocultures. Reliance on a partner has been proven to be sufficient to slow the rate of adaptation through a mathematical model (Adamowicz et al. 2020). Ye et al. found that the asymmetric metabolic adaptations could also undermine the stability of a cross‐feeding consortium of two amino acid auxotrophic *Escherichia coli* strains during evolution. Through dynamic metabolic assays, the authors found that the lysine auxotrophic strain displayed metabolic plasticity, adjusting lysine utilisation in response to lysine availability, while the arginine auxotrophic strain lacked similar plasticity. Then, the former had the ability to outcompete the latter and dominate the consortium, resulting in collapse (Ye et al. 2025).

## 4 Impacts of Cross‐Feeding on Strain Evolution

**FIGURE 3:**
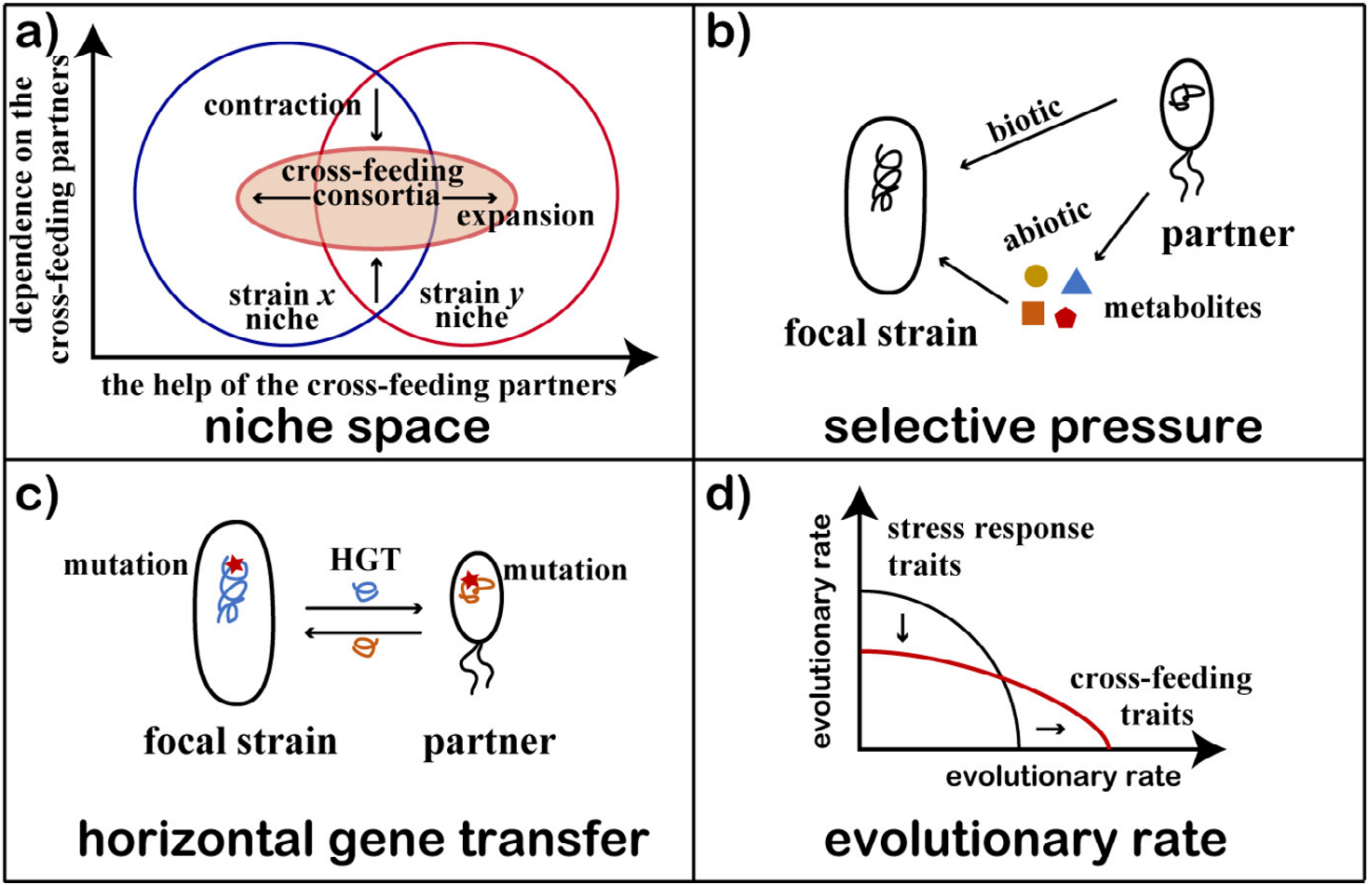
The impacts of cross‐feeding on focal strain evolution around four aspects: (a) niche space. The niche space of cross‐feeding consortia can be expanded due to the help of the partners, but contracted due to the dependence on the partners. (b) selective pressure. Partners can act as biotic factors directly or change the abiotic environment by secreting metabolites, affecting the selective pressures of the focal strains. (c) horizontal gene transfer. Partners with different metabolic abilities bring functional genes into the consortia, providing the opportunity for horizontal gene transfer. (d) evolutionary rate. Cross‐feeding changes the selective pressures and then affects the evolutionary rates. The constant selective pressures for cross‐feeding may increase the evolutionary rates of cross‐feeding traits, but obligate cross‐feeding seems to slow the evolutionary rates of strains to respond to changing environmental conditions.

The evolution of cross‐feeding consortia differs from that of single strains. Interactions with partners may affect the evolutionary trajectories of the focal strains. Interacting partners can take up space, consume nutrients, secrete metabolites, and form direct cell‐to‐cell connections with focal strains. Understanding how interactions shape evolution is important to predict community dynamics, especially in complex natural communities consisting of countless species (Barraclough 2015). Here, we briefly generalise the impacts of cross‐feeding on strain evolution from four aspects: niche space, selective pressure, horizontal gene transfer, and evolutionary rate (Figure 3). This will help to understand the changes of cross‐feeding in the evolutionary process due to the presence of interacting partners.

### 4.1 Niche Space

Niche space is the ecological and evolutionary foundation that abstractly summarises the environmental conditions where strains can survive (Malard and Guisan 2023). The presence of interacting partners can change the niche space of the focal strain compared with the monoculture (Matthews et al. 2014). For example, in a cross‐feeding consortium, strains can grow in an environment where an essential nutrient is lacking because of the supplement from their partners. In this case, the niche space of the focal strain is expanded due to the metabolite exchange (Oña et al. 2021). Moreover, cross‐feeding can also expand niche space by promoting tolerance or persistence when facing stresses such as antibiotics or heavy metals (Wang et al. 2024; Xiong et al. 2024). However, the cross‐feeding strains survive depending on their partners due to the coupled metabolic pathway. They simultaneously face environmental pressure that previously only constrained one of them. The “weakest‐link hypothesis” provides a good example that when species rely on each other for essential metabolites, the minimum inhibitory concentration (MIC) of the consortia may drop to that of the “weakest link”, the strains least resistant in monoculture (Adamowicz et al. 2018). Then, the niche space of the focal strain is contracted.

### 4.2 Selective Pressure

Selective pressure is the driver of strain evolution and may be affected by the presence of cross‐feeding partners or even the different proportions of strains. Partners can act as biotic factors directly or change the abiotic environment by secreting metabolites (Semenec et al. 2023; Zuchowski et al. 2023). Then, changed environments may have different selective pressures on strains, thus affecting the evolutionary trajectories of the focal strains by determining which mutants can be kept. For example, the costly galactose secretion was selected for because it increased the growth of the cross‐feeding partners, bringing more production of methionine in return. The abiotic and biotic factors were both important for the selection of galactose secretion (Harcombe et al. 2018). Dolinšek et al. established a cross‐feeding *Pseudomonas stutzeri* consortium consisting of a generalist that could reduce nitrate to nitrogen gas and a specialist that could only reduce nitrite to nitrogen gas. Interestingly, the authors found that the initial ratio of specialist to generalist controlled the phenotypic diversification and trajectory of the generalist. High initial generalist frequencies meant that nitrite remained available to the generalist after nitrate was completely consumed, leading to an increased selective pressure for generalist phenotypes with improved nitrite reduction (Dolinšek et al. 2022).

### 4.3 Horizontal Gene Transfer

The mutation is the original approach to acquiring new genotypes and is the only approach for monoculture. However, when genotypes with different metabolic abilities coexist, different functional genes are brought into the consortia. The focal strains can obtain functional genes through a new approach, horizontal gene transfer (HGT) (Goldman and Kaçar 2022; Kehila et al. 2023). Through mobile genetic elements, functional genes can be transferred from one strain to another. Such gene flow plays an important role in shaping community function and microbial evolution (Arnold et al. 2022; Haudiquet et al. 2022). In a cross‐feeding consortium, different functional genes are carried by different phenotypes, and the functional complementation brings fitness benefits. Niche specialisation may create barriers to HGT because genes that are beneficial to a prototroph may not be beneficial to a closely related auxotroph and vice versa, but the gene flow between ecological populations still exists, no matter how marked the decline (Shapiro et al. 2012). The strains can regain the genes that they have lost and shift from cross‐feeding to autonomy through HGT if the environmental conditions are more suitable for autonomy and allow HGT to occur (Chen et al. 2024).

### 4.4 Evolutionary Rate

Interactions can affect the evolutionary ability of strains to adapt to environmental changes, and experiments have repeatedly demonstrated that mutualism does alter the evolutionary rates of strains (Lilja and Johnson 2019; Adamowicz et al. 2020; Pauli et al. 2022). However, the specific effects are different. In some cases, increased evolutionary rates occurred due to the coevolution with constantly changing selective pressures. In other cases, mutualism favoured the more slowly evolving partner and therefore decreased the rate of evolution (Chacón et al. 2021). For example, the coevolution of auxotrophic phenotypes in coculture enhanced the rate of adaptation relative to the two control groups that were capable of independent growth (Preussger et al. 2020). Alternatively, obligate cooperative mutualism could constrain the evolutionary ability of strains to respond to changing environmental conditions (Pauli et al. 2022). In both rifampicin and ampicillin treatments, it was also observed that resistance evolved more slowly in obligate cocultures of *Escherichia coli* and *Salmonella enterica* than in monocultures (Adamowicz et al. 2020). The varied impacts of cross‐feeding on evolutionary rates are likely due to a trade‐off between the evolution of different traits. The improved evolutionary rates of metabolic traits by cross‐feeding may be at the expense of the evolutionary ability of other traits, such as antibiotic resistance.

## 5 Concluding Remarks

Ecological and evolutionary processes can influence the structure and function of microbial consortia over comparable timescales (Martiny et al. 2023). Interaction between strains can affect the evolutionary process; interactions may change when the strains evolve new traits, forming eco‐evolutionary feedbacks (Andrade‐Domínguez et al. 2014). Thus, it is worth combining the ecological and evolutionary processes to understand microbial consortia. The interaction keeps changing from an evolutionary perspective, especially for microbes. To better construct cross‐feeding consortia, an overall evolutionary understanding is needed, including the coevolutionary history of strains before construction, the environmental conditions selecting for cross‐feeding, and the evolutionary directions of cross‐feeding after formation that we reviewed here. Strains with coevolutionary history are more likely to be cooperative than competitive because of niche differentiation (Pastore et al. 2021). They may adapt to cross‐feeding more quickly than engineered strains without any coevolutionary process. Also, microbial consortia under conditions with higher selective pressures for cross‐feeding may convert to this mutualistic interaction more easily. However, quantifying the direction and intensity of selective pressures in particular environmental conditions remains challenging, limiting the ability to predict the evolutionary direction and speed. After cross‐feeding formation, microbial consortia may collapse if the conditions select against cross‐feeding. So, only by fully understanding evolutionary processes can functional synthetic microbial consortia based on cross‐feeding be constructed efficiently and be long‐lasting.

In this review, we described the features of two opposite evolutionary directions after cross‐feeding formation (Table 1). The stronger metabolic coupling, deeper growth dependence, and deeper evolutionary dependence between cross‐feeders indicate that cross‐feeding is strengthened when conditions favour adaptation to the mutualistic lifestyle. On the contrary, unsuitable environmental conditions can lead to the direct collapse of the cross‐feeding consortia due to metabolic decoupling, partner extinction, or cheater dominance. The loss of the fitness advantage and the constraints on the evolutionary ability can also lead to the weakening of cross‐feeding. The uncertain evolutionary direction reminds us that an effective evaluation method for mapping a detailed genotype‐fitness landscape is still lacking. Bridging genotypes and strain fitness in different lifestyles is still hard in certain environmental parameter sets. The shape of the fitness landscape is also sensitive to environmental changes (Chen et al. 2022). In brief, the strains tend to climb the peak in the fitness landscape and evolve to a higher fitness position. However, knowing what environmental factors are involved and how certain parameters affect the fitness landscape remains a challenge. For example, the unexpected purine cross‐feeding (LaSarre et al. 2020; Chuang et al. 2024), vitamin and amino acid cross‐feeding (Martinson et al. 2023) shows how easy it is to overlook cross‐feeding interactions due to the lack of knowledge of what conditions are suitable for cross‐feeding before experiments. Maybe an effort to summarise the most decisive factor or a comprehensive workflow including several factors to determine the strain fitness can help predict the evolutionary dynamics. Then, we can know in which conditions cross‐feeding can occur and in which conditions the strains in cross‐feeding consortia will shift to competitive or autonomous genotypes.

**TABLE 1:** Summary of the features of the evolutionary directions of cross‐feeding after formation.

Types	Performances	Mechanisms	Refs
Strengthening			
Stronger metabolic coupling	– Increased secretion of metabolites for exchange	– More benefits in return from partners – Comparative advantage and the economies of scale	Konstantinidis et al. (2021) Harcombe (2010) Adkins‐Jablonsky et al. (2021)
	– Coupling of broader metabolic pathways	– Further gene loss reducing costs – New metabolite secretion increasing net benefits	D'Souza and Kost (2016) Wang et al. (2021) Harcombe et al. (2018)
Deeper growth dependence	– Better growth with partners	– Mutations contributing to growth in coculture were selected	Zhang and Reed (2014) Zuchowski et al. (2023)
	– More dependence on partners	– The costly adaptation to partners needed enough return – Rewired metabolic network for mutual lifestyle	Zhang and Reed (2014) Preussger et al. (2020) Scarinci et al. (2024)
Deeper evolutionary dependence	– Different evolutionary states affected by partners	– Different selected mutations due to the influence of cross‐feeding partners when coculturing or responding to stress	Harcombe et al. (2018) Zuchowski et al. (2023) Adamowicz et al. (2020)
Weakening			
Collapse of the interaction consortia	– The metabolite coupling disappeared	– Metabolites from the nutrient‐rich environment rather than partners – Bypass or regain the lost function	Murillo‐Roos et al. (2022) Germerodt et al. (2016) Melero‐Jiménez et al. (2025) Chen et al. (2024)
	– The extinction of partners	– Too small population size – Unsuitable environmental conditions	Sanchez and Gore (2013) Ratzke and Gore (2018) Ratzke et al. (2018)
	– The challenge of cheaters	– Overexploitation of shared metabolites by cheaters	Strassmann et al. (2000) Velicer et al. (2000)
Loss of the fitness advantage	– The metabolic strategy shifts from cross‐feeding to autonomy or others	– Changed fitness landscape due to changed environmental conditions	Chen et al. (2024) Melero‐Jiménez et al. (2025)
Constraints on the evolutionary ability	– Limited evolutionary ability of cross‐feeding consortia	– Decreased evolutionary rates of strains – asymmetric evolutionary abilities between the interacting strains	Pauli et al. (2022) Adamowicz et al. (2020) Ye et al. (2025)

Most cross‐feeding studies are based on experimental consortia where the members are known. However, natural microbial communities consist of numerous strains lacking genetic information, forming a complex interaction network (Faust and Raes 2012). Cross‐feeding is a subset of the interaction network, and the cross‐feeding consortium can be seen as a subcommunity in microbial communities. There is still a knowledge gap between the cross‐feeding subcommunity and the whole community. On the one hand, the cross‐feeding subcommunity affects the community's diversity, stability, and productivity. Cross‐feeding provides an exometabolome that can help strains survive with less reliance on abiotic environments, and the functional specialisation seems to improve the whole production efficiency. From this aspect, cross‐feeding may improve community diversity, stability, and productivity (Yang et al. 2020). However, the cross‐feeders are auxotrophs that cannot survive without external metabolites because they have lost their essential metabolic functions. Secreted metabolites may become energy waste if the absorption and utilisation are blocked. In this case, cross‐feeding impairs the stability and productivity of the community (Oliveira et al. 2014). On the other hand, community members also affect the cross‐feeding subcommunity. Other strains in the community can change the abiotic and biotic environments of the cross‐feeding subcommunity, affecting the evolutionary process (Westley et al. 2024). They can form different interactions with cross‐feeders and even higher‐order interactions (Gibbs et al. 2024), weakening the interactions among the members of the subcommunity. So, there are still challenges to getting a full picture of cross‐feeding in a complex community context.

## Author Contributions

**Laipeng Luo:** writing – original draft, visualization. **Xiaoli Chen:** writing – review and editing, methodology. **Bingwen Liu:** writing – review and editing, visualization. **Yong Nie:** writing – review and editing, methodology, supervision. **Xiao‐Lei Wu:** writing – review and editing, supervision, funding acquisition.

## Conflicts of Interest

The authors declare no conflicts of interest.

## Data Availability

Data sharing is not applicable to this article as no new data were created or analyzed in this study.

## References

[emi470175-bib-0001] Adamowicz, E. M. , J. Flynn , R. C. Hunter , and W. R. Harcombe . 2018. “Cross‐Feeding Modulates Antibiotic Tolerance in Bacterial Communities.” ISME Journal 12: 2723–2735. 29991761 10.1038/s41396-018-0212-z PMC6194032

[emi470175-bib-0002] Adamowicz, E. M. , and W. R. Harcombe . 2020. “Weakest‐Link Dynamics Predict Apparent Antibiotic Interactions in a Model Cross‐Feeding Community.” Antimicrobial Agents and Chemotherapy 64: e00465‐20. 32778550 10.1128/AAC.00465-20 PMC7577160

[emi470175-bib-0003] Adamowicz, E. M. , M. Muza , J. M. Chacon , and W. R. Harcombe . 2020. “Cross‐Feeding Modulates the Rate and Mechanism of Antibiotic Resistance Evolution in a Model Microbial Community of *Escherichia Coli* and *Salmonella enterica* .” PLoS Pathogens 16: e1008700. 32687537 10.1371/journal.ppat.1008700 PMC7392344

[emi470175-bib-0004] Adkins‐Jablonsky, S. J. , C. M. Clark , S. E. Papoulis , M. D. Kuhl , and J. J. Morris . 2021. “Market Forces Determine the Distribution of a Leaky Function in a Simple Microbial Community.” Proceedings of the National Academy of Sciences of the United States of America 118: e2109813118. 34548403 10.1073/pnas.2109813118 PMC8488675

[emi470175-bib-0005] Andrade‐Domínguez, A. , E. Salazar , M. del Carmen Vargas‐Lagunas , R. Kolter , and S. Encarnación . 2014. “Eco‐Evolutionary Feedbacks Drive Species Interactions.” ISME Journal 8: 1041–1054. 24304674 10.1038/ismej.2013.208 PMC3996687

[emi470175-bib-0006] Arnold, B. J. , I. T. Huang , and W. P. Hanage . 2022. “Horizontal Gene Transfer and Adaptive Evolution in Bacteria.” Nature Reviews Microbiology 20: 206–218. 34773098 10.1038/s41579-021-00650-4

[emi470175-bib-0007] Aulakh, S. K. , L. Selles Vidal , E. J. South , et al. 2023. “Spontaneously Established Syntrophic Yeast Communities Improve Bioproduction.” Nature Chemical Biology 19: 951–961. 37248413 10.1038/s41589-023-01341-2 PMC10374442

[emi470175-bib-0008] Barraclough, T. G. 2015. “How Do Species Interactions Affect Evolutionary Dynamics Across Whole Communities?” Annual Review of Ecology, Evolution, and Systematics 46: 25–48.

[emi470175-bib-0009] Bottery, M. J. , J. W. Pitchford , and V.‐P. Friman . 2021. “Ecology and Evolution of Antimicrobial Resistance in Bacterial Communities.” ISME Journal 15: 939–948. 33219299 10.1038/s41396-020-00832-7 PMC8115348

[emi470175-bib-0010] Bull, J. J. , and W. R. Harcombe . 2009. “Population Dynamics Constrain the Cooperative Evolution of Cross‐Feeding.” PLoS One 4: e4115. 19127304 10.1371/journal.pone.0004115 PMC2614108

[emi470175-bib-0011] Carr, E. C. , S. D. Harris , J. R. Herr , and W. R. Riekhof . 2021. “Lichens and Biofilms: Common Collective Growth Imparts Similar Developmental Strategies.” Algal Research 54: 102217.

[emi470175-bib-0012] Chacón, J. M. , S. P. Hammarlund , J. N. V. Martinson , L. B. Smith , and W. R. Harcombe . 2021. “The Ecology and Evolution of Model Microbial Mutualisms.” Annual Review of Ecology, Evolution, and Systematics 52: 363–384.

[emi470175-bib-0013] Chen, J. Z. , D. M. Fowler , and N. Tokuriki . 2022. “Environmental Selection and Epistasis in an Empirical Phenotype‐Environment‐Fitness Landscape.” Nature Ecology & Evolution 6: 427–438. 35210579 10.1038/s41559-022-01675-5

[emi470175-bib-0014] Chen, X. , M. Wang , L. Luo , et al. 2024. “The Evolution of Autonomy From Two Cooperative Specialists in Fluctuating Environments.” Proceedings of the National Academy of Sciences of the United States of America 121: e2317182121. 39172793 10.1073/pnas.2317182121 PMC11363282

[emi470175-bib-0015] Chomicki, G. , E. T. Kiers , and S. S. Renner . 2020. “The Evolution of Mutualistic Dependence.” Annual Review of Ecology, Evolution, and Systematics 51: 409–432.

[emi470175-bib-0016] Chuang, Y.‐C. , N. W. Haas , R. Pepin , et al. 2024. “Bacterial Adenine Cross‐Feeding Stems From a Purine Salvage Bottleneck.” ISME Journal 18: wrae034. 38452196 10.1093/ismejo/wrae034 PMC10976475

[emi470175-bib-0017] de Visser, J. A. G. M. , and J. Krug . 2014. “Empirical Fitness Landscapes and the Predictability of Evolution.” Nature Reviews Genetics 15: 480–490. 10.1038/nrg3744 24913663

[emi470175-bib-0018] Dolinšek, J. , J. Ramoneda , and D. R. Johnson . 2022. “Initial Community Composition Determines the Long‐Term Dynamics of a Microbial Cross‐Feeding Interaction by Modulating Niche Availability.” ISME Communications 2: 1–10. 37938324 10.1038/s43705-022-00160-1 PMC9723679

[emi470175-bib-0019] Douglas, A. E. 2020. “The Microbial Exometabolome: Ecological Resource and Architect of Microbial Communities.” Philosophical Transactions of the Royal Society, B: Biological Sciences 375: 20190250. 10.1098/rstb.2019.0250 PMC7133521 32200747

[emi470175-bib-0020] Dragos, A. , M. Martin , C. Falcon Garcia , et al. 2018. “Collapse of Genetic Division of Labour and Evolution of Autonomy in Pellicle Biofilms.” Nature Microbiology 3: 1451–1460. 10.1038/s41564-018-0263-y 30297741

[emi470175-bib-0021] D'Souza, G. , and C. Kost . 2016. “Experimental Evolution of Metabolic Dependency in Bacteria.” PLoS Genetics 12: e1006364. 27814362 10.1371/journal.pgen.1006364 PMC5096674

[emi470175-bib-0022] D'Souza, G. , J. Schwartzman , J. Keegstra , et al. 2023. “Interspecies Interactions Determine Growth Dynamics of Biopolymer‐Degrading Populations in Microbial Communities.” Proceedings of the National Academy of Sciences of the United States of America 120: e2305198120. 37878716 10.1073/pnas.2305198120 PMC10622921

[emi470175-bib-0023] D'Souza, G. , S. Shitut , D. Preussger , G. Yousif , S. Waschina , and C. Kost . 2018. “Ecology and Evolution of Metabolic Cross‐Feeding Interactions in Bacteria.” Natural Product Reports 35: 455–488. 29799048 10.1039/c8np00009c

[emi470175-bib-0024] D'Souza, G. , S. Waschina , C. Kaleta , and C. Kost . 2015. “Plasticity and Epistasis Strongly Affect Bacterial Fitness After Losing Multiple Metabolic Genes.” Evolution 69: 1244–1254. 25765095 10.1111/evo.12640

[emi470175-bib-0025] D'Souza, G. , S. Waschina , S. Pande , K. Bohl , C. Kaleta , and C. Kost . 2014. “Less Is More: Selective Advantages Can Explain the Prevalent Loss of Biosynthetic Genes in Bacteria.” Evolution 68: 2559–2570. 24910088 10.1111/evo.12468

[emi470175-bib-0026] Durand, R. , J. Jalbert‐Ross , A. Fijarczyk , A. K. Dubé , and C. R. Landry . 2023. “Cross‐Feeding Affects the Target of Resistance Evolution to an Antifungal Drug.” PLoS Genetics 19: e1011002. 37856537 10.1371/journal.pgen.1011002 PMC10617708

[emi470175-bib-0027] Dutta, D. , and S. Saini . 2021. “Cell Growth Model With Stochastic Gene Expression Helps Understand the Growth Advantage of Metabolic Exchange and Auxotrophy.” MSystems 6: e00448‐21. 34342540 10.1128/mSystems.00448-21 PMC8407474

[emi470175-bib-0028] Faust, K. , and J. Raes . 2012. “Microbial Interactions: From Networks to Models.” Nature Reviews Microbiology 10: 538–550. 22796884 10.1038/nrmicro2832

[emi470175-bib-0029] Finn, D. R. , M. App , L. Hertzog , and C. C. Tebbe . 2022. “Reconciling Concepts of Black Queen and Tragedy of the Commons in Simulated Bulk Soil and Rhizosphere Prokaryote Communities.” Frontiers in Microbiology 13: 969784. 36187971 10.3389/fmicb.2022.969784 PMC9520196

[emi470175-bib-0030] Fragata, I. , A. Blanckaert , M. A. Dias Louro , D. A. Liberles , and C. Bank . 2019. “Evolution in the Light of Fitness Landscape Theory.” Trends in Ecology & Evolution 34: 69–82. 30583805 10.1016/j.tree.2018.10.009

[emi470175-bib-0031] Fritts, R. K. , J. T. Bird , M. G. Behringer , et al. 2020. “Enhanced Nutrient Uptake Is Sufficient to Drive Emergent Cross‐Feeding Between Bacteria in a Synthetic Community.” ISME Journal 14: 2816–2828. 32788711 10.1038/s41396-020-00737-5 PMC7784955

[emi470175-bib-0032] Ge, Z.‐B. , Z.‐Q. Zhai , W.‐Y. Xie , et al. 2023. “Two‐Tiered Mutualism Improves Survival and Competitiveness of Cross‐Feeding Soil Bacteria.” ISME Journal 17: 2090–2102. 37737252 10.1038/s41396-023-01519-5 PMC10579247

[emi470175-bib-0033] Germerodt, S. , K. Bohl , A. Luck , et al. 2016. “Pervasive Selection for Cooperative Cross‐Feeding in Bacterial Communities.” PLoS Computational Biology 12: e1004986. 27314840 10.1371/journal.pcbi.1004986 PMC4912067

[emi470175-bib-0034] Ghoul, M. , A. S. Griffin , and S. A. West . 2014. “Toward an Evolutionary Definition of Cheating.” Evolution 68: 318–331. 24131102 10.1111/evo.12266

[emi470175-bib-0035] Gibbs, T. L. , G. Gellner , S. A. Levin , K. S. McCann , A. Hastings , and J. M. Levine . 2024. “When Can Higher‐Order Interactions Produce Stable Coexistence?” Ecology Letters 27: e14458. 38877741 10.1111/ele.14458

[emi470175-bib-0036] Giri, S. , G. Yousif , S. Shitut , L. Oña , and C. Kost . 2022. “Prevalent Emergence of Reciprocity Among Cross‐Feeding Bacteria.” ISME Communications 2: 1–7. 37938764 10.1038/s43705-022-00155-y PMC9723789

[emi470175-bib-0037] Goldman, A. D. , and B. Kaçar . 2022. “Very Early Evolution From the Perspective of Microbial Ecology.” Environmental Microbiology 25: 5–10. 35944516 10.1111/1462-2920.16144

[emi470175-bib-0038] Gorter, F. A. , M. Manhart , and M. Ackermann . 2020. “Understanding the Evolution of Interspecies Interactions in Microbial Communities.” Philosophical Transactions of the Royal Society, B: Biological Sciences 375: 20190256. 10.1098/rstb.2019.0256 PMC7133538 32200743

[emi470175-bib-0039] Hammarlund, S. P. , J. M. Chacon , and W. R. Harcombe . 2019. “A Shared Limiting Resource Leads to Competitive Exclusion in a Cross‐Feeding System.” Environmental Microbiology 21: 759–771. 30507059 10.1111/1462-2920.14493 PMC6634945

[emi470175-bib-0040] Harcombe, W. 2010. “Novel Cooperation Experimentally Evolved Between Species.” Evolution 64: 2166–2172. 20100214 10.1111/j.1558-5646.2010.00959.x

[emi470175-bib-0041] Harcombe, W. R. , J. M. Chacon , E. M. Adamowicz , L. M. Chubiz , and C. J. Marx . 2018. “Evolution of Bidirectional Costly Mutualism From Byproduct Consumption.” Proceedings of the National Academy of Sciences of the United States of America 115: 12000–12004. 30348787 10.1073/pnas.1810949115 PMC6255176

[emi470175-bib-0042] Haudiquet, M. , J. M. de Sousa , M. Touchon , and E. P. C. Rocha . 2022. “Selfish, Promiscuous and Sometimes Useful: How Mobile Genetic Elements Drive Horizontal Gene Transfer in Microbial Populations.” Philosophical Transactions of the Royal Society, B: Biological Sciences 377: 20210234. 10.1098/rstb.2021.0234 PMC9393566 35989606

[emi470175-bib-0043] Hillesland, K. L. , and D. A. Stahl . 2010. “Rapid Evolution of Stability and Productivity at the Origin of a Microbial Mutualism.” Proceedings of the National Academy of Sciences of the United States of America 107: 2124–2129. 20133857 10.1073/pnas.0908456107 PMC2836651

[emi470175-bib-0044] Hu, B. , M. X. Wang , S. Geng , et al. 2020. “Metabolic Exchange With Non‐Alkane‐Consuming *Pseudomonas Stutzeri* SLG510A3‐8 Improves *n*‐Alkane Biodegradation by the Alkane Degrader *Dietzia* sp. Strain DQ12‐45‐1b.” Applied and Environmental Microbiology 86: e02931‐19. 32033953 10.1128/AEM.02931-19 PMC7117941

[emi470175-bib-0045] Huelsmann, M. , O. T. Schubert , and M. Ackermann . 2024. “A Framework for Understanding Collective Microbiome Metabolism.” Nature Microbiology 9: 3097–3109. 10.1038/s41564-024-01850-3 39604625

[emi470175-bib-0046] Kehila, D. , K. T. C. Wong , and N. Tokuriki . 2023. “Evolution of New Metabolic Pathways and Microbial Communities.” Current Opinion in Systems Biology 36: 100472.

[emi470175-bib-0047] Konstantinidis, D. , F. Pereira , E. M. Geissen , et al. 2021. “Adaptive Laboratory Evolution of Microbial Co‐Cultures for Improved Metabolite Secretion.” Molecular Systems Biology 17: e10189. 34370382 10.15252/msb.202010189 PMC8351387

[emi470175-bib-0048] Kost, C. , K. R. Patil , J. Friedman , S. L. Garcia , and M. Ralser . 2023. “Metabolic Exchanges are Ubiquitous in Natural Microbial Communities.” Nature Microbiology 8: 2244–2252. 10.1038/s41564-023-01511-x 37996708

[emi470175-bib-0049] LaSarre, B. , A. M. Deutschbauer , C. E. Love , and J. B. McKinlay . 2020. “Covert Cross‐Feeding Revealed by Genome‐Wide Analysis of Fitness Determinants in a Synthetic Bacterial Mutualism.” Applied and Environmental Microbiology 86: e00543‐20. 32332139 10.1128/AEM.00543-20 PMC7301861

[emi470175-bib-0050] LaSarre, B. , A. L. McCully , J. T. Lennon , and J. B. McKinlay . 2017. “Microbial Mutualism Dynamics Governed by Dose‐Dependent Toxicity of Cross‐Fed Nutrients.” ISME Journal 11: 337–348. 27898053 10.1038/ismej.2016.141 PMC5270580

[emi470175-bib-0051] Lawrence, D. , F. Fiegna , V. Behrends , et al. 2012. “Species Interactions Alter Evolutionary Responses to a Novel Environment.” PLoS Biology 10: e1001330. 22615541 10.1371/journal.pbio.1001330 PMC3352820

[emi470175-bib-0052] Lee, J. A. , A. C. Baugh , N. J. Shevalier , B. Strand , S. Stolyar , and C. J. Marx . 2021. “Cross‐Feeding of a Toxic Metabolite in a Synthetic Lignocellulose‐Degrading Microbial Community.” Microorganisms 9: 321. 33557371 10.3390/microorganisms9020321 PMC7914493

[emi470175-bib-0053] Libby, E. , and W. C. Ratcliff . 2021. “Lichens and Microbial Syntrophies Offer Models for an Interdependent Route to Multicellularity.” Lichenologist 53: 283–290.

[emi470175-bib-0054] Lilja, E. E. , and D. R. Johnson . 2019. “Substrate Cross‐Feeding Affects the Speed and Trajectory of Molecular Evolution Within a Synthetic Microbial Assemblage.” BMC Evolutionary Biology 19: 129. 31221104 10.1186/s12862-019-1458-4 PMC6584980

[emi470175-bib-0055] Lindsay, R. J. , B. J. Pawlowska , and I. Gudelj . 2019. “Privatization of Public Goods Can Cause Population Decline.” Nature Ecology & Evolution 3: 1206–1216. 31332334 10.1038/s41559-019-0944-9

[emi470175-bib-0056] Liu, Z. B. , H. Huang , M. B. Qi , X. J. Wang , O. O. Adebanjo , and Z. M. Lu . 2019. “Metabolite Cross‐Feeding Between *Rhodococcus Ruber* YYL and *Bacillus Cereus* MLY1 in the Biodegradation of Tetrahydrofuran Under pH Stress.” Applied and Environmental Microbiology 85: e01196‐19. 31375492 10.1128/AEM.01196-19 PMC6752023

[emi470175-bib-0057] Lopez, J. G. , and N. S. Wingreen . 2022. “Noisy Metabolism Can Promote Microbial Cross‐Feeding.” eLife 11: e70694. 35380535 10.7554/eLife.70694 PMC8983042

[emi470175-bib-0058] Lundh, T. , and P. Gerlee . 2013. “Cross‐Feeding Dynamics Described by a Series Expansion of the Replicator Equation.” Bulletin of Mathematical Biology 75: 709–724. 23494900 10.1007/s11538-013-9828-3

[emi470175-bib-0059] Ma, Z. , M. Jiang , C. Liu , et al. 2024. “Quinolone‐Mediated Metabolic Cross‐Feeding Develops Aluminium Tolerance in Soil Microbial Consortia.” Nature Communications 15: 10148. 10.1038/s41467-024-54616-0 PMC11584702 39578460

[emi470175-bib-0060] Machado, D. , O. M. Maistrenko , S. Andrejev , et al. 2021. “Polarization of Microbial Communities Between Competitive and Cooperative Metabolism.” Nature Ecology & Evolution 5: 195–203. 33398106 10.1038/s41559-020-01353-4 PMC7610595

[emi470175-bib-0061] Malard, L. A. , and A. Guisan . 2023. “Into the Microbial Niche.” Trends in Ecology & Evolution 38: 936–945. 37236880 10.1016/j.tree.2023.04.015

[emi470175-bib-0062] Marchal, M. , F. Goldschmidt , S. N. Derksen‐Müller , S. Panke , M. Ackermann , and D. R. Johnson . 2017. “A Passive Mutualistic Interaction Promotes the Evolution of Spatial Structure Within Microbial Populations.” BMC Evolutionary Biology 17: 106. 28438135 10.1186/s12862-017-0950-y PMC5402672

[emi470175-bib-0063] Martinson, J. N. V. , J. M. Chacón , B. A. Smith , A. R. Villarreal , R. C. Hunter , and W. R. Harcombe . 2023. “Mutualism Reduces the Severity of Gene Disruptions in Predictable Ways Across Microbial Communities.” ISME Journal 17: 2270–2278. 37865718 10.1038/s41396-023-01534-6 PMC10689784

[emi470175-bib-0064] Martiny, J. B. H. , A. C. Martiny , E. Brodie , et al. 2023. “Investigating the Eco‐Evolutionary Response of Microbiomes to Environmental Change.” Ecology Letters 26: S81–S90. 36965002 10.1111/ele.14209

[emi470175-bib-0065] Mataigne, V. , N. Vannier , P. Vandenkoornhuyse , and S. Hacquard . 2021. “Microbial Systems Ecology to Understand Cross‐Feeding in Microbiomes.” Frontiers in Microbiology 12: 780469. 34987488 10.3389/fmicb.2021.780469 PMC8721230

[emi470175-bib-0066] Matthews, B. , L. De Meester , C. G. Jones , et al. 2014. “Under Niche Construction: An Operational Bridge Between Ecology, Evolution, and Ecosystem Science.” Ecological Monographs 84: 245–263.

[emi470175-bib-0067] Mavrommati, M. , A. Daskalaki , S. Papanikolaou , and G. Aggelis . 2022. “Adaptive Laboratory Evolution Principles and Applications in Industrial Biotechnology.” Biotechnology Advances 54: 107795. 34246744 10.1016/j.biotechadv.2021.107795

[emi470175-bib-0068] McKinlay, J. B. 2023. “Are Bacteria Leaky? Mechanisms of Metabolite Externalization in Bacterial Cross‐Feeding.” Annual Review of Microbiology 77: 277–297. 10.1146/annurev-micro-032521-023815 37285553

[emi470175-bib-0069] Meijer, J. , B. van Dijk , and P. Hogeweg . 2020. “Contingent Evolution of Alternative Metabolic Network Topologies Determines Whether Cross‐Feeding Evolves.” Communications Biology 3: 401. 32728180 10.1038/s42003-020-1107-x PMC7391776

[emi470175-bib-0070] Melero‐Jiménez, I. J. , Y. Sorokin , A. Merlin , J. Li , A. Couce , and J. Friedman . 2025. “Mutualism Breakdown Underpins Evolutionary Rescue in an Obligate Cross‐Feeding Bacterial Consortium.” Nature Communications 16: 3482. 10.1038/s41467-025-58742-1 PMC11992082 40216843

[emi470175-bib-0071] Morris, J. J. , R. E. Lenski , and E. R. Zinser . 2012. “The Black Queen Hypothesis: Evolution of Dependencies Through Adaptive Gene Loss.” MBio 3: e00036‐12. 22448042 10.1128/mBio.00036-12 PMC3315703

[emi470175-bib-0072] Murillo‐Roos, M. , H. S. M. Abdullah , M. Debbar , N. Ueberschaar , and M. T. Agler . 2022. “Cross‐Feeding Niches Among Commensal Leaf Bacteria Are Shaped by the Interaction of Strain‐Level Diversity and Resource Availability.” ISME Journal 16: 2280–2289. 35768644 10.1038/s41396-022-01271-2 PMC9381498

[emi470175-bib-0073] Oliveira, N. M. , R. Niehus , and K. R. Foster . 2014. “Evolutionary Limits to Cooperation in Microbial Communities.” Proceedings of the National Academy of Sciences of the United States of America 111: 17941–17946. 25453102 10.1073/pnas.1412673111 PMC4273359

[emi470175-bib-0074] Oña, L. , S. Giri , N. Avermann , M. Kreienbaum , K. M. Thormann , and C. Kost . 2021. “Obligate Cross‐Feeding Expands the Metabolic Niche of Bacteria.” Nature Ecology & Evolution 5: 1224–1232. 34267366 10.1038/s41559-021-01505-0

[emi470175-bib-0075] Pande, S. , H. Merker , K. Bohl , et al. 2014. “Fitness and Stability of Obligate Cross‐Feeding Interactions That Emerge Upon Gene Loss in Bacteria.” ISME Journal 8: 953–962. 24285359 10.1038/ismej.2013.211 PMC3996690

[emi470175-bib-0076] Pande, S. , S. Shitut , L. Freund , et al. 2015. “Metabolic Cross‐Feeding via Intercellular Nanotubes Among Bacteria.” Nature Communications 6: 6238. 10.1038/ncomms7238 25703793

[emi470175-bib-0077] Pastore, A. I. , G. Barabas , M. D. Bimler , M. M. Mayfield , and T. E. Miller . 2021. “The Evolution of Niche Overlap and Competitive Differences.” Nature Ecology & Evolution 5: 330–337. 33495591 10.1038/s41559-020-01383-y

[emi470175-bib-0078] Pauli, B. , L. Ona , M. Hermann , and C. Kost . 2022. “Obligate Mutualistic Cooperation Limits Evolvability.” Nature Communications 13: 337. 10.1038/s41467-021-27630-9 PMC8764027 35039522

[emi470175-bib-0079] Pearl Mizrahi, S. , A. Goyal , and J. Gore . 2023. “Community Interactions Drive the Evolution of Antibiotic Tolerance in Bacteria.” Proceedings of the National Academy of Sciences of the United States of America 120: e2209043119. 36634144 10.1073/pnas.2209043119 PMC9934204

[emi470175-bib-0080] Piccardi, P. , E. Ulrich , M. Garcia‐Garcerà , R. D. Martino , S. E. A. Testa , and S. Mitri . 2024. “The Evolution of Reduced Facilitation in a Four‐Species Bacterial Community.” Evolution Letters 8: 1–840. 39677578 10.1093/evlett/qrae036 PMC11637553

[emi470175-bib-0081] Pontrelli, S. , R. Szabo , S. Pollak , et al. 2022. “Metabolic Cross‐Feeding Structures the Assembly of Polysaccharide Degrading Communities.” Science Advances 8: eabk3076. 35196097 10.1126/sciadv.abk3076 PMC8865766

[emi470175-bib-0082] Preussger, D. , S. Giri , L. K. Muhsal , L. Ona , and C. Kost . 2020. “Reciprocal Fitness Feedbacks Promote the Evolution of Mutualistic Cooperation.” Current Biology 30: 3580–3590. 32707067 10.1016/j.cub.2020.06.100

[emi470175-bib-0083] Rafiqi, A. , A. Rajakumar , and E. Abouheif . 2020. “Origin and Elaboration of a Major Evolutionary Transition in Individuality.” Nature 585: 239–244. 32879485 10.1038/s41586-020-2653-6

[emi470175-bib-0084] Ratzke, C. , J. Denk , and J. Gore . 2018. “Ecological Suicide in Microbes.” Nature Ecology & Evolution 2: 867–872. 29662223 10.1038/s41559-018-0535-1 PMC5911225

[emi470175-bib-0085] Ratzke, C. , and J. Gore . 2018. “Modifying and Reacting to the Environmental pH Can Drive Bacterial Interactions.” PLoS Biology 16: e2004248. 29538378 10.1371/journal.pbio.2004248 PMC5868856

[emi470175-bib-0086] Salazar, A. , and S. Mitri . 2025. “Can a Microbial Community Become an Evolutionary Individual?” Current Opinion in Microbiology 84: 102596. 39983253 10.1016/j.mib.2025.102596

[emi470175-bib-0087] San Roman, M. , and A. Wagner . 2020. “Acetate and Glycerol Are Not Uniquely Suited for the Evolution of Cross‐Feeding in *E. coli* .” PLoS Computational Biology 16: e1008433. 33253183 10.1371/journal.pcbi.1008433 PMC7728234

[emi470175-bib-0088] Sanchez, A. , and J. Gore . 2013. “Feedback Between Population and Evolutionary Dynamics Determines the Fate of Social Microbial Populations.” PLoS Biology 11: e1001547. 23637571 10.1371/journal.pbio.1001547 PMC3640081

[emi470175-bib-0089] Scarinci, G. , J.‐L. Ariens , G. Angelidou , et al. 2024. “Enhanced Metabolic Entanglement Emerges During the Evolution of an Interkingdom Microbial Community.” Nature Communications 15: 7238. 10.1038/s41467-024-51702-1 PMC11341674 39174531

[emi470175-bib-0090] Scheuerl, T. , M. Hopkins , R. W. Nowell , D. W. Rivett , T. G. Barraclough , and T. Bell . 2020. “Bacterial Adaptation Is Constrained in Complex Communities.” Nature Communications 11: 754. 10.1038/s41467-020-14570-z PMC7005322 32029713

[emi470175-bib-0091] Semenec, L. , A. K. Cain , C. J. Dawson , et al. 2023. “Cross‐Protection and Cross‐Feeding Between *Klebsiella Pneumoniae* and *Acinetobacter baumannii* Promotes Their Co‐Existence.” Nature Communications 14: 702. 10.1038/s41467-023-36252-2 PMC9911699 36759602

[emi470175-bib-0092] Sexton, D. J. , and M. Schuster . 2017. “Nutrient Limitation Determines the Fitness of Cheaters in Bacterial Siderophore Cooperation.” Nature Communications 8: 230. 10.1038/s41467-017-00222-2 PMC5550491 28794499

[emi470175-bib-0093] Sgobba, E. , and V. F. Wendisch . 2020. “Synthetic Microbial Consortia for Small Molecule Production.” Current Opinion in Biotechnology 62: 72–79. 31627138 10.1016/j.copbio.2019.09.011

[emi470175-bib-0094] Shapiro, B. J. , J. Friedman , O. X. Cordero , et al. 2012. “Population Genomics of Early Events in the Ecological Differentiation of Bacteria.” Science 336: 48–51. 22491847 10.1126/science.1218198 PMC3337212

[emi470175-bib-0095] Silverstein, M. R. , J. M. Bhatnagar , and D. Segre . 2024. “Metabolic Complexity Drives Divergence in Microbial Communities.” Nature Ecology & Evolution 8: 1493–1504.38956426 10.1038/s41559-024-02440-6

[emi470175-bib-0096] Smith, N. W. , P. R. Shorten , E. Altermann , N. C. Roy , and W. C. McNabb . 2019. “The Classification and Evolution of Bacterial Cross‐Feeding.” Frontiers in Ecology and Evolution 7: 153.

[emi470175-bib-0097] Smith, P. , and M. Schuster . 2024. “The Fitness Benefit of Pyoverdine Cross‐Feeding by *Pseudomonas Protegens* Pf‐5.” Environmental Microbiology 26: e16554. 38097191 10.1111/1462-2920.16554

[emi470175-bib-0098] Strassmann, J. E. , Y. Zhu , and D. C. Queller . 2000. “Altruism and Social Cheating in the Social Amoeba *Dictyostelium Discoideum* .” Nature 408: 965–967. 11140681 10.1038/35050087

[emi470175-bib-0099] Stump, S. M. , E. C. Johnson , and C. A. Klausmeier . 2018. “Local Interactions and Self‐Organized Spatial Patterns Stabilize Microbial Cross‐Feeding Against Cheaters.” Journal of the Royal Society Interface 15: 20170822. 29563243 10.1098/rsif.2017.0822 PMC5908524

[emi470175-bib-0100] Sun, Z. , T. Koffel , S. M. Stump , G. M. Grimaud , and C. A. Klausmeier . 2019. “Microbial Cross‐Feeding Promotes Multiple Stable States and Species Coexistence, but Also Susceptibility to Cheaters.” Journal of Theoretical Biology 465: 63–77. 30639296 10.1016/j.jtbi.2019.01.009

[emi470175-bib-0101] Szathmary, E. 2015. “Toward Major Evolutionary Transitions Theory 2.0.” Proceedings of the National Academy of Sciences of the United States of America 112: 10104–10111. 25838283 10.1073/pnas.1421398112 PMC4547294

[emi470175-bib-0102] Tasoff, J. , M. T. Mee , and H. H. Wang . 2015. “An Economic Framework of Microbial Trade.” PLoS One 10: e0132907. 26222307 10.1371/journal.pone.0132907 PMC4519184

[emi470175-bib-0103] Van den Bergh, B. , T. Swings , M. Fauvart , and J. Michiels . 2018. “Experimental Design, Population Dynamics, and Diversity in Microbial Experimental Evolution.” Microbiology and Molecular Biology Reviews 82: e00008‐18. 30045954 10.1128/MMBR.00008-18 PMC6094045

[emi470175-bib-0104] van Tatenhove‐Pel, R. J. , D. H. de Groot , A. S. Bisseswar , B. Teusink , and H. Bachmann . 2021. “Population Dynamics of Microbial Cross‐Feeding Are Determined by Co‐Localization Probabilities and Cooperation‐Independent Cheater Growth.” ISME Journal 15: 3050–3061. 33953364 10.1038/s41396-021-00986-y PMC8443577

[emi470175-bib-0105] Velicer, G. J. , L. Kroos , and R. E. Lenski . 2000. “Developmental Cheating in the Social Bacterium *Myxococcus xanthus* .” Nature 404: 598–601. 10766241 10.1038/35007066

[emi470175-bib-0106] Venkataram, S. , H.‐Y. Kuo , E. F. Y. Hom , and S. Kryazhimskiy . 2023. “Mutualism‐Enhancing Mutations Dominate Early Adaptation in a Two‐Species Microbial Community.” Nature Ecology & Evolution 7: 143–154. 36593292 10.1038/s41559-022-01923-8

[emi470175-bib-0107] Vet, S. , L. Gelens , and D. Gonze . 2020. “Mutualistic Cross‐Feeding in Microbial Systems Generates Bistability via an Allee Effect.” Scientific Reports 10: 7763. 32385386 10.1038/s41598-020-63772-4 PMC7210978

[emi470175-bib-0108] Vidal, M. C. , and K. A. Segraves . 2021. “Coevolved Mutualists Experience Fluctuating Costs and Benefits Over Time.” Evolution 75: 219–230. 33368192 10.1111/evo.14155

[emi470175-bib-0109] Wang, M. , X. Liu , Y. Nie , and X. L. Wu . 2021. “Selfishness Driving Reductive Evolution Shapes Interdependent Patterns in Spatially Structured Microbial Communities.” ISME Journal 15: 1387–1401. 33343001 10.1038/s41396-020-00858-x PMC8115099

[emi470175-bib-0110] Wang, X. , N. Guo , Y. Zhang , G. Wang , and K. Shi . 2024. “Cross‐Protection and Cross‐Feeding Between *Enterobacter* and *Comamonas* Promoting Their Coexistence and Cadmium Tolerance in *Oryza sativa* L.” Microbiological Research 286: 127806. 38924817 10.1016/j.micres.2024.127806

[emi470175-bib-0111] Weinbach, A. , N. Loeuille , and R. P. Rohr . 2022. “Eco‐Evolutionary Dynamics Further Weakens Mutualistic Interaction and Coexistence Under Population Decline.” Evolutionary Ecology 36: 373–387.

[emi470175-bib-0112] Werner, G. D. , J. E. Strassmann , A. B. Ivens , et al. 2014. “Evolution of Microbial Markets.” Proceedings of the National Academy of Sciences of the United States of America 111: 1237–1244. 24474743 10.1073/pnas.1315980111 PMC3910570

[emi470175-bib-0113] West, S. A. , R. M. Fisher , A. Gardner , and E. T. Kiers . 2015. “Major Evolutionary Transitions in Individuality.” Proceedings of the National Academy of Sciences of the United States of America 112: 10112–10119. 25964342 10.1073/pnas.1421402112 PMC4547252

[emi470175-bib-0114] Westley, J. , F. C. García , R. Warfield , and G. Yvon‐Durocher . 2024. “The Community Background Alters the Evolution of Thermal Performance.” Evolution Letters 8: 505–513. 39100233 10.1093/evlett/qrae007 PMC11291943

[emi470175-bib-0115] Xiong, X. Y. , H. G. Othmer , and W. R. Harcombe . 2024. “Emergent Antibiotic Persistence in a Spatially Structured Synthetic Microbial Mutualism.” ISME Journal 18: wrae075. 38691424 10.1093/ismejo/wrae075 PMC11104777

[emi470175-bib-0116] Yang, D. D. , A. Alexander , M. Kinnersley , et al. 2020. “Fitness and Productivity Increase With Ecotypic Diversity Among *Escherichia Coli* Strains That Coevolved in a Simple, Constant Environment.” Applied and Environmental Microbiology 86: e00051‐20. 32060029 10.1128/AEM.00051-20 PMC7117940

[emi470175-bib-0117] Ye, N. , Z. C. Yang , and Z. D. Bai . 2025. “Asymmetric Metabolic Adaptations Undermine Stability in Microbial Syntrophy.” ISME Communications 5: ycaf011. 39944238 10.1093/ismeco/ycaf011 PMC11815887

[emi470175-bib-0118] Zachar, I. , and G. Boza . 2020. “Endosymbiosis Before Eukaryotes: Mitochondrial Establishment in Protoeukaryotes.” Cellular and Molecular Life Sciences 77: 3503–3523. 32008087 10.1007/s00018-020-03462-6 PMC7452879

[emi470175-bib-0119] Zhang, X. , and J. L. Reed . 2014. “Adaptive Evolution of Synthetic Cooperating Communities Improves Growth Performance.” PLoS One 9: e108297. 25299364 10.1371/journal.pone.0108297 PMC4191979

[emi470175-bib-0120] Zhao, Y. , Y. Feng , J. Zhou , et al. 2023. “Potential Bacterial Isolation by Dosing Metabolites in Cross‐Feedings.” Water Research 231: 119589. 36645941 10.1016/j.watres.2023.119589

[emi470175-bib-0121] Zhu, M. L. , and X. F. Dai . 2024. “Shaping of Microbial Phenotypes by Trade‐Offs.” Nature Communications 15: 4238. 10.1038/s41467-024-48591-9 PMC11102524 38762599

[emi470175-bib-0122] Zuchowski, R. , S. Schito , F. Neuheuser , et al. 2023. “Discovery of Novel Amino Acid Production Traits by Evolution of Synthetic Co‐Cultures.” Microbial Cell Factories 22: 71. 37061714 10.1186/s12934-023-02078-2 PMC10105947

